# Cost-Effectiveness of an Absorbable Antibacterial Envelope for Infection Control in Cardiac Implantable Electronic Device Procedures in Spain

**DOI:** 10.36469/001c.156166

**Published:** 2026-02-06

**Authors:** Tomás Datino, Daniela Afonso, Elana Greaves, Simon Eggington, Julen Monje, María Álvarez Orozco, Claudia Wolff, Stuart Mealing, Arístides de Alarcón

**Affiliations:** 1 Universidade Complutense de Madrid; 2 York Health Economics Consortium, York, UK; 3 Medtronic International Trading Sàrl, Tolochenaz, Switzerland; 4 Medtronic Ibérica, Madrid, Spain; 5 Clinical Unit of Infectious Diseases, Microbiology, and Parasitology, Virgen del Rocío University Hospital, Seville, Spain

**Keywords:** cardiac implantable electronic device, infection prevention, absorbable antibacterial envelope, cost-effectiveness, value for money, high risk

## Abstract

**Background:**

Infections represent the most serious complication associated with cardiac implantable electronic devices (CIEDs). This can result in prolonged hospital stays, high morbidity and mortality, and a significant economic burden for healthcare systems.

**Objectives:**

This study aimed to evaluate the cost-effectiveness of the TYRX absorbable antibacterial envelope for CIED infection prevention from the Spanish Healthcare System perspective.

**Methods:**

A decision tree model with a lifetime horizon was developed to compare standard antibiotic prophylaxis with its combination with TYRX, regardless of infection risk. The model incorporated infection incidence, mortality, and utility values up to 36 months, derived from REINFORCE, AdaptResponse, and WRAP-IT studies. Unit costs (2025 euros) included prevention strategies and infection management. Lifetime costs and quality-adjusted life-years (QALYs) were assigned to survivors beyond 36 months. The incremental cost-effectiveness ratio (ICER) was reported by CIED and weighted by implant distribution (permanent pacemaker [PPM, 76.5%], implantable cardioverter-defibrillator [ICD, 15.2%], cardiac resynchronization therapy with defibrillator [CRT-D, 5.4%], and pacemaker [CRT-P, 2.9%]). A subgroup analysis was performed in high-risk patients (PADIT≥7), modifying infection rates based on PADIT risk stratification, along with sensitivity analyses. Model inputs were validated by an expert panel.

**Results:**

TYRX was the dominant strategy (more effective and less costly) for CRT-D and ICD recipients and cost-effective for those receiving PPM (€17 740/QALY) or CRT-P (€14 647/QALY), considering a willingness-to-pay threshold of €25 000/QALY. Across the spectrum of CIEDs, the ICER was €11 709/QALY. TYRX remained cost-effective in 77% of sensitivity analysis simulations. In high-risk patients, TYRX was dominant for all CIEDs.

**Discussion:**

This study is believed to be the first economic evaluation of TYRX in Spain and provides novel evidence in a broad, unselected population. Previous cost-effectiveness analyses conducted across different healthcare systems have consistently shown that TYRX is cost-effective in patients at elevated risk for device-related infections. Although the populations and healthcare settings differ, our findings are consistent with this body of evidence.

**Conclusions:**

TYRX represents a dominant strategy for infection prevention for CRT-D and ICD and is cost-effective for PPM and CRT-P, based on Spain’s willingness to pay.

## INTRODUCTION

Cardiac implantable electronic devices (CIEDs) are well-established treatments for a variety of cardiac arrhythmias.^1^ Although CIEDs are an effective and safe therapy, serious complications still occur.^2–4^

CIED-related infections are one of the most serious device complications and are associated with prolonged hospital stays and high morbidity and mortality, representing a significant economic burden for the healthcare system. In Spain, the cost of CIED-related infections has been estimated at €34 086 for systemic infections and €21 790 for local infections, with mortality rates of 10.8% and 2.5%, respectively.^5^

The current standard of care (SOC) to prevent CIED-related infections involves prophylactic antibiotic administration.^6,7^ Despite this preventive measure, the incidence of infections is estimated to range between 1% and 4%, with a rising trend primarily attributed to the growing use of more complex devices in older patients with multiple comorbidities.^8^ If an infection occurs, the current treatment protocol is based around device extraction, antibiotic therapy, and, ideally, reimplantation.^9^

TYRX (Medtronic, Inc.) is a single-use absorbable antibacterial envelope designed to stabilize the device pocket. It contains the antibiotics rifampicin and minocycline, which are gradually released over the course of 7 days.^10^ The clinical efficacy of TYRX has been demonstrated in high-quality clinical trials and real-world studies. The World-wide Randomized Antibiotic Envelope Infection Prevention (WRAP-IT) trial, a large, prospective, randomized, controlled, multicenter study, showed a 40% relative risk (RR) reduction in major device-related infections at 12 months with the use of TYRX compared with SOC.^11^ These findings were further supported by the REINFORCE study, a real-world observational analysis conducted in a European setting, which confirmed a consistent reduction in infection rates across various CIED and individual risk profiles.^12^

The use of TYRX is likely to increase healthcare costs in the short term because it is used adjunctively to SOC, but this could be balanced by infection management savings. While TYRX has been shown to be cost-effective in several European countries, there are currently no studies exploring its economic impact in Spain.^1,13,14^

Although there is an increasing widespread use of the envelope in high-risk patients, its adoption in other populations is still limited, potentially due to acquisition costs. Thus, it is important to explore the economic benefits of using TYRX to determine the feasibility of adopting such technology in healthcare settings with limited resources. The present analysis aimed to explore the cost-effectiveness of TYRX in the prevention of post-CIED implantation infection in Spain.

## METHODS

### Model Structure

**Figure 1.**
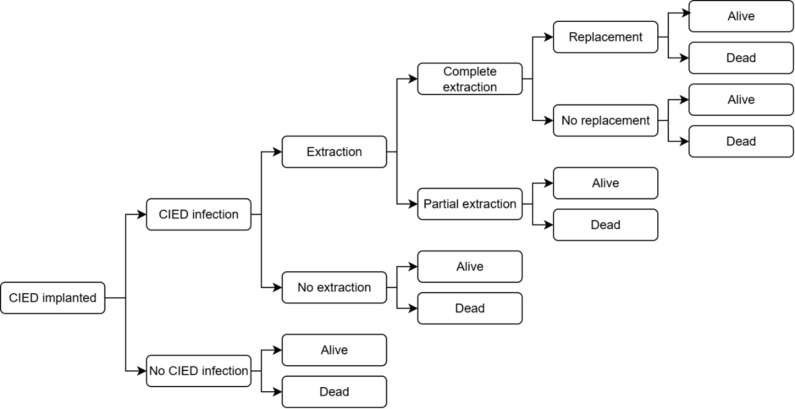
Decision Tree Model Abbreviation: CIED, cardiac implantable electronic device.

A previously developed and published decision tree model was adapted to represent the clinical pathway of patients undergoing CIED implantation (**Figure 1**).^1,13,14^ Costs and benefits, expressed as quality-adjusted life-years (QALYs) and life-years (LYs), were calculated over a lifetime horizon and compared between patients receiving TYRX + SOC and those receiving SOC alone, regardless of infection risk (broad, unselected population). This study was conducted from the perspective of the Spanish National Health System.

At the beginning of the decision tree, all patients were at risk of post-implantation infection. Individuals who experienced an infection had either a complete or partial extraction (with or without a device replacement) or no device extraction. Infection management strategies were the same in both arms of the model. Death and survival at 36 months were end nodes for all arms of the decision tree, and lifetime cost and QALY “payoff” values were used for the remainder of the individual’s life expectancy.

Four CIED types were included in the analysis. The incremental cost-effectiveness ratio (ICER) was calculated, both stratified by device type and weighted, based on the distribution of the diverse implants reported in Spain by national registries: permanent pacemaker (PPM, 76.5%), implantable cardioverter-defibrillator (ICD, 15.2%), cardiac resynchronization therapy either with defibrillator (CRT-D, 5.4%), or pacemaker (CRT-P, 2.9%).^15,16^

Although there is no officially established cost-effectiveness threshold in Spain, an intervention is usually considered cost-effective when the ICER is below €25 000/QALY gained.^17^ Moreover, when a strategy is as or more effective and less costly than the alternative option, it is considered to be a dominant strategy.^18^

**Table 1** shows the key model inputs used in the base case analysis.

**Table 1.** Model Parameters

**Parameter**	**Value**	**Source**
Proportion of patients with each device, %		
CRT-D	5.39	Asensi et al (2024),^15^ Molina-Lerma, et al (2023)^16^
CRT-P	2.94	
PPM	76.46	
ICD	15.21	
Probability of extraction after infection, %		
Extractions undertaken	100	WRAP-IT trial^11^
Complete	86.10	
Complete with replacement	96.00	
Partial	13.9	
Probability of major CIED infection with standard of care (all devices), %		
0-12 mo	2.75	REINFORCE^12^
12-24 mo	0.90	
24-36 mo	0.83	
Hazard ratio of major CIED infection with TYRX (all devices)		
0-12, 12-24, and 24-36 mo	0.28	REINFORCE^12^
Scenario analysis (high-risk population): Probability of infection, standard of care, %		
0-12 mo	7.87	REINFORCE,^12^ De Heide et al (2024)^19^
12-24 mo	2.58	
24-36 mo	2.37	
Probability of relapse, %		
No extraction	71.40	Pichlmaier et al (2011)^20^
Partial extraction	50.00	Chua et al (2000)^21^
Complete extraction (with replacement)	4.80	Ahmed et al (2019)^22^
Probability of minor CIED infection		
Standard of care, %	0.95	Kay, et al (2018)^13^
Relative risk with TYRX	0.76	
Mortality at 36 mo (all devices)		
No infection, %	9.50	AdaptResponse^23^
Hazard ratio with infection	2.3	WRAP-IT trial^11^
Cost of infection prevention per patient, €		
Standard of care	7.89	Expert opinion, General Council of Pharmacists^24^
TYRX	1200	Data on file^25^
Cost of infection prevent, unit drug cost, price per mg, €		
Cefazolin	0.0010	General Council of Pharmacists^24^
Vancomicin	0.0057	General Council of Pharmacists^24^
Teicoplanin	0.0538	General Council of Pharmacists^24^
Cloxacillin	0.0011	General Council of Pharmacists^24^
Total cost of infection, complete extraction and replacement, €		
CRT-D	46 979	Egea et al (2018),^26^ Maceira-Rozas et al (2018),^27^ data on file,^25^ expert opinion
CRT-P	32 904	
PPM	30 960	
ICD	43 569	
Total cost of infection, complete or partial extraction with no replacement, €		
All devices	27 605	Egea et al (2018),^26^ data on file,^25^ expert opinion
Total cost of infection, no extraction, €		
All devices	23 486	Egea et al (2018),^26^ data on file,^25^ expert opinion
Total cost of minor CIED infection, €		
All devices	3664	Oblikue^28^
Lifetime costs, €		
Without CIED		
CRT-D	11 194	NICE TA314^29^
CRT-P	11 194	
PPM	11 194	
ICD	23 358	
With CIED		
CRT-D	29 861	NICE TA314^29^
CRT-P	26 722	
PPM	26 722	
ICD	23 716	
Utility		
CRT-D	0.81	Boriani et al (2021),^1^ Kay et al (2018),^13^ Frausing et al (2023)^14^
CRT-P	0.76	
PPM	0.81	
ICD	0.84	
Disutility applied for major CIED infections/relapse (applied for 6 mo)	−0.10	
Lifetime QALYs		
Without CIED		
CRT-D	3.48	NICE TA314^29^
CRT-P	3.48	
PPM	3.48	
ICD	5.95	
With CIED		
CRT-D	4.58	NICE TA314^29^
CRT-P	4.17	
PPM	4.17	
ICD	6.75	

Abbreviations: CIED, cardiac implantable electronic device; CRT-D, cardiac resynchronization therapy with defibrillator; CRT-P, cardiac resynchronization therapy with pacemaker; ICD, implantable cardioverter-defibrillator; PPM, permanent pacemaker; QALY, quality-adjusted life-year.

### Key Clinical Data Sources

The WRAP-IT, REINFORCE, and AdaptResponse studies were used to populate the model.^11,12,23^ The WRAP-IT study randomized 6893 patients with an increased risk of CIED infection across 25 countries.^11^ The REINFORCE study enrolled 1819 patients who had received an initial CIED implant or were due to undergo a CIED surgery.^12^ The AdaptResponse study, which compared clinical outcomes of adaptive vs conventional CRT in heart failure patients, involved 227 hospitals across 27 countries, including several in Europe.^23^

### Infection Risks/Probability of Infection

A major infection is defined as an infection that results in a CIED removal, an invasive CIED procedure without removal, treatment with long-term antibiotic therapy if extraction is not possible, or death.^11^ The probability of major infection with SOC was estimated from the control arm of the propensity-matched population in the REINFORCE study, with the same value considered for all device types: 2.75%, 0.90%, and 0.83% at 12, 12 to 24, and 24 to 36 months, respectively.^12^ The treatment effect associated with TYRX was included by applying a hazard ratio (HR) of 0.28, obtained from the same propensity-matched population, and applied uniformly across all device types.^12^ Relapse rates were sourced from the literature, with a risk of 71.4% in people without an extraction, 50.0% with partial extraction, and 4.80% with complete extraction and replacement.^20–22^ Individuals who had a complete extraction without replacement were assumed to have no risk of relapse due to the absence of an implanted device.

A minor CIED infection was defined as any infection not meeting the criteria for a major CIED infection.^11^ A probability of minor CIED infection of 0.95% was included for those receiving SOC, applying a RR of 0.76 to those in the TYRX + SOC arm.^13^ The values were assumed to be the same across device types.

### Mortality

All-cause mortality data up to 36 months was 9.5% for those without an infection, derived from the AdaptResponse study.^23^ An HR of 2.3, derived from the WRAP-IT trial, was applied to those with an infection, resulting in a 3-year probability of death of 20.5%.^11^

### Health-Related Quality of Life

Baseline health-related quality-of-life values were based on EQ-5D-3L data from WRAP-IT.^1,13,14^ Baseline utilities after CIED implantation were different between device types (CRT-D, 0.81; CRT-P, 0.76; PPM, 0.81; ICD, 0.84). A utility decrement of 0.10 was applied for 6 months to patients who experienced an infection, regardless of device type or whether TYRX was used.^1,13,14^

### Costs and Resource Use

In line with the perspective adopted, only direct healthcare costs were considered in the analysis. All costs are expressed in euros valued as of 2025.

Regarding the prevention strategy, the cost of TYRX was €1200.^25^ Costs associated with prophylactic antibiotics were applied as a one-time cost each time an individual received a CIED. The proportion of use per individual for each drug, as well as the dosage in milligrams per course, was defined and validated by an expert panel. Unit costs for each drug were obtained from a national pharmacological cost database.^24^

For patients who experienced a major infection, the costs of extraction, replacement, hospital stay, and antibiotic treatment were estimated based on a national study.^26^ In the case of minor infections, unit cost was derived by averaging unitary tariffs identified through a national database that collects health costs from different sources.^28^ For specific resources such as temporary pacing, wearable defibrillators, and leadless devices, resource use was obtained from the expert panel.

Device costs were sourced from a national health technology assessment (HTA) report, and internal unpublished procurement data reflecting the national average across all implants in Spain.^25,27^

### Lifetime Costs and QALY

Beyond 36 months, discounted lifetime costs and QALYs were assigned to survivors, based on estimates from the National Institute for Health and Care Excellence’s (NICE) Evidence Review Group (TA314).^29^ These were dependent on whether a CIED was in place and the device type. The health benefits calculated within the initial 3-year time horizon were subtracted from the lifetime estimates, as these apply to the entire cohort and not only to those still alive at that point. Similarly, the initial procedure cost was excluded from lifetime costs. All costs were converted from British pounds to euros, using an exchange rate of 1.19, and updated to 2025 values, accounting for both currency conversion and inflation.^30^

### Discount Rate

A discount rate of 3.0% was applied to costs and QALYs in years 2 and 3, in line with Spanish pharmacoeconomic guidelines.^31^

### Expert Panel

The model structure and all input values were validated and agreed upon by a panel consisting of a cardiologist and an infectious disease specialist. Structured prework material was developed with all the parameters identified in the literature that were proposed for use in the model. This document was reviewed individually by the experts, and subsequently, two consensus meetings were held to validate and agree on values and assumptions where needed.

### Sensitivity Analysis

One-way deterministic sensitivity analyses were conducted to establish first-order uncertainty around infection rates, mortality, utility, unit costs, lifetime QALYs and costs, and time horizon, and to evaluate their impact on incremental net benefit, described as the increase in effectiveness multiplied by the willingness to pay per unit of effectiveness (expressed in monetary terms) minus the increase in cost, yielding the incremental net benefit. Input parameters were varied using associated SD or 95% confidence intervals, if provided. All other parameters were adjusted by ±20%.

Probabilistic sensitivity analyses were conducted using 10 000 iterations. Beta distributions were used for probabilities and utilities, gamma for costs, and lognormal for HR. Uncertainty estimates were sourced from the literature where available. Where data were not available, standard errors of 10% to 20% of the mean were used.

### Scenario Analysis

An alternative scenario was conducted to evaluate outcomes in a high-risk population, defined as patients with a PADIT score of at least 7, by adjusting infection rates based on PADIT risk stratification. The RR of infection for high-risk patients compared with the general population was first calculated as 2.87, based on data from a retrospective study by de Heide et al.^19^ Subsequently, the infection rates used in the base case (broad, unselected population) were multiplied by this RR to estimate the infection probabilities for high-risk patients (12 months, 7.87%; 12-24 months, 2.58%; 24-36 months, 2.37%). This approach was used because no Kaplan-Meier curves showing infection incidence over time for the PADIT subgroups were available in the REINFORCE study. Finally, for the TYRX + SOC arm, the HR of 0.28 reported in the REINFORCE study was applied to these adjusted rates.^12^

## RESULTS

### Base Case

Over a lifetime horizon, TYRX decreased the absolute rate of major CIED infection, device extraction, and hospitalization each by 71.8%, and mortality by 3.5% relative to SOC.

**Table 2** presents the deterministic cost-effectiveness results stratified by device type. For CRT-D and ICD populations, TYRX was estimated to save €97 and €106 per person and generate an additional 0.02 and 0.03 QALYs, and 0.04 and 0.03 LYs, respectively.

**Table 2.** Deterministic Base Case Results Stratified by Device Type

**Treatment**	**TYRX + SOC**	**SOC**	**Incremental**
Mixed			
Cost per person, €	25 553.79	25 326.83	226.96
QALYs per person	4.35	4.33	0.02
LYs per person	5.69	5.66	0.02
**Incremental cost-effectiveness ratio, €/QALY**			11 708.53
**Incremental cost-effectiveness ratio, €/LY**			9314.31
**Incremental net benefit, €**			257.64
CRT-D			
Cost per person, €	28 781.88	28 878.38	−96.50
QALYs per person	4.34	4.32	0.02
LYs per person	6.76	6.73	0.04
**Incremental cost-effectiveness ratio, €/QALY**			Dominant
**Incremental cost-effectiveness ratio, €/LY**			Dominant
**Incremental net benefit, €**			621.85
CRT-P			
Cost per person, €	25 799.19	25 538.96	260.23
QALYs per person	3.96	3.94	0.02
LYs per person	5.23	5.21	0.02
**Incremental cost-effectiveness ratio, €/QALY**			14 647.06
**Incremental cost-effectiveness ratio, €/LY**			11 843.48
**Incremental net benefit, €**			183.94
PPM			
Cost per person, €	25 780.04	25 465.22	314.81
QALYs per person	3.97	3.95	0.02
LYs per person	5.23	5.21	0.02
**Incremental cost-effectiveness ratio, €/QALY**			17 740.12
**Incremental cost-effectiveness ratio, €/LY**			14 327.79
**Incremental net benefit, €**			128.83
ICD			
Cost per person, €	23 225.09	23 331.58	−106.49
QALYs per person	6.31	6.28	0.03
LYs per person	7.70	7.67	0.03
**Incremental cost-effectiveness ratio, €/QALY**			Dominant
**Incremental cost-effectiveness ratio, €/LY**			Dominant
**Incremental net benefit, €**			790.35

Abbreviations: CRT-D, cardiac resynchronization therapy with defibrillator; CRT-P, cardiac resynchronization therapy with pacemaker; ICD, implantable cardioverter-defibrillator; LY, life-year; PPM, permanent pacemaker; QALY, quality-adjusted life year; SOC, standard of care.

Individuals who received either CRT-P or PPM were estimated to incur additional costs of €260 and €315 per person, yielding 0.02 and 0.02 QALYs, and 0.02 and 0.02 LYs, respectively. These equated to ICERs of €14 647/QALY and €17 740/QALY gained, and €11 843/LY and €14 328/LY gained for CRT-P and PPM, respectively.

In the mixed population, TYRX incurred an incremental cost of €227 while generating an additional 0.02 QALYs and 0.02 LYs per person, with an ICER of €11 709/QALY gained and €9314/LY gained.

Based on these results, TYRX was considered a dominant option in patients with CRT-D and ICD (ie, it is more effective and less costly)and a cost-effective strategy for those implanted with CRT-P and PPM, with ICERs below the generally accepted willingness-to-pay threshold in Spain.^17^ Considering the mixed population, TYRX remained cost-effective, supporting its value across a broad range of CIED.

### Sensitivity Analysis

**Figure 2.**
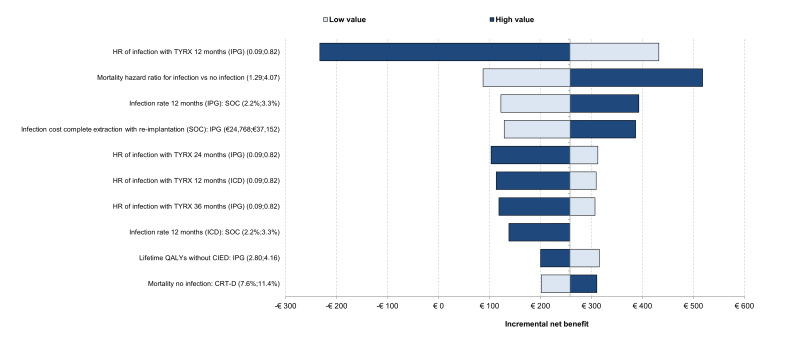
Tornado Diagram Abbreviations: CIED, cardiac implantable electronic device; CRT-D, cardiac resynchronization therapy with defibrillator; HR, hazard ratio; ICD, implantable cardioverter-defibrillator; PPM, permanent pacemaker; SOC, standard of care. One-way sensitivity analyses showing the effect of varying model parameters on the estimated incremental net benefit. The effects of the lower and the higher parameter range are represented in different colors.

The univariate analysis for the mixed population is presented in **Figure 2**. The main driver of the results was the PPM infection HR of TYRX at 12 months. The mortality HR for infection vs no infection, infection rate at 12 months in SOC for PPM, and the infection cost for complete extraction with reimplantation for PPM were also key drivers of the results. Only the changes to the infection HR at 12 months caused TYRX to become not cost-effective.

**Figure 3.**
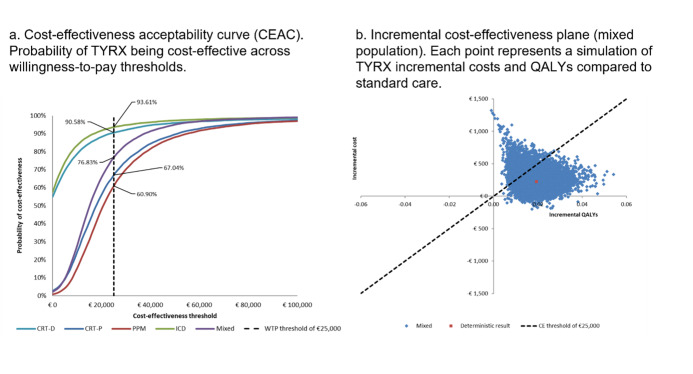
Cost-Effectiveness Acceptability Curve (**A**) and Incremental Cost-Effectiveness Plane (**B**) for the Mixed Population Abbreviations: CE, cost-effectiveness; CRT-D, cardiac resynchronization therapy defibrillator; CRT-P, cardiac resynchronization therapy pacemaker ; ICD, implantable cardioverter defibrillator; PPM, permanent pacemaker; QALY, quality-adjusted life year; WTP, willingness-to-pay. Each line represents the probability of being cost-effective across different willingness-to-pay thresholds. Each point represents a simulation of TYRX incremental costs and QALYs compared with standard care.

At a cost-effectiveness threshold of €25 000/QALY gained, the probabilistic sensitivity analysis results indicated that the probability of TYRX was likely to be cost-effective for those with CRT-D, CRT-P, PPM, ICD, and mixed population was 91%, 67%, 61%, 94% and 77%, respectively. The cost-effectiveness plane for the mixed population is shown in **Figure 3.**

### Scenario Analysis

In the high-risk cohort, TYRX was associated with cost savings of approximately €2474 for CRT-D, €1458 for CRT-P, €1303 for PPM, and €2502 for ICD, resulting in overall savings of €1553 per patient (**Table 3**). Additionally, TYRX generated gains in QALYs and LYs across all device types, with increases of 0.06 QALYs and 0.1 LYs for CRT-D, 0.05 QALYs and 0.06 LYs for CRT-P, 0.05 QALYs and 0.06 LYs for PPM, and 0.08 QALYs and 0.09 LYs for ICD, leading to overall gains of 0.05 QALYs and 0.07 LYs. Thus, TYRX was a dominant strategy for all devices in this population.

**Table 3.** Scenario Analysis (High-Risk Population) Results Stratified by Device Type

**Treatment**	**TYRX + SOC**	**SOC**	**Incremental**
Mixed			
Cost per person, €	26 309.54	27 862.62	−1553.08
QALYs per person	4.33	4.28	0.05
LYs per person	5.67	5.60	0.07
**Incremental cost-effectiveness ratio, €/QALY**			Dominant
**Incremental cost-effectiveness ratio, €/LY**			Dominant
**Incremental net benefit, €**			2903.34
CRT-D			
Cost per person, €	29 779.72	32 253.42	−2473.70
QALYs per person	4.32	4.27	0.06
LYs per person	6.73	6.64	0.10
**Incremental cost-effectiveness ratio, €/QALY**			Dominant
**Incremental cost-effectiveness ratio, €/LY**			Dominant
**Incremental net benefit, €**			3940.06
CRT-P			
Cost per person, €	26 525.82	27 984.30	−1458.48
QALYs per person	3.94	3.89	0.05
LYs per person	5.21	5.15	0.06
**Incremental cost-effectiveness ratio, €/QALY**			Dominant
**Incremental cost-effectiveness ratio, €/LY**			Dominant
**Incremental net benefit, €**			2695.47
PPM			
Cost per person, €	26 470.20	27 773.22	−1303.02
QALYs per person	3.96	3.91	0.05
LYs per person	5.21	5.15	0.06
**Incremental cost-effectiveness ratio, €/QALY**			Dominant
**Incremental cost-effectiveness ratio, €/LY**			Dominant
**Incremental net benefit, €**			2536.81
ICD			
Cost per person, €	24 230.38	26 732.52	−2502.14
QALYs per person	6.29	6.21	0.08
LYs per person	7.68	7.59	0.09
**Incremental cost-effectiveness ratio, €/QALY**			Dominant
**Incremental cost-effectiveness ratio, €/LY**			Dominant
**Incremental net benefit, €**			4418.65

Abbreviations: CRT-D, cardiac resynchronization therapy with defibrillator; CRT-P, cardiac resynchronization therapy with pacemaker; ICD, implantable cardioverter-defibrillator; LY, life-year; PPM, permanent pacemaker; QALY, quality-adjusted life-year; SOC, standard of care.

## DISCUSSION

An estimated 56 040 patients receive a CIED each year in Spain.^15,16^ Although the incidence of CIED-related infections is relatively low, the associated clinical and economic burden is substantial.^32^ In this context, preventive strategies aimed at reducing infection risk are of particular importance. Despite the demonstrated clinical benefits, the adoption of TYRX to date remains limited, especially among patients who are not classified as high-risk.^10^ This restrained uptake may be primarily due to concerns over the up-front acquisition cost, which can be perceived as a barrier to routine use in lower-risk populations. This study fills a critical gap by providing up-to-date, country-specific evidence on the cost-effectiveness of TYRX within the Spanish healthcare system and offers valuable insights for healthcare providers and policymakers, facilitating more informed decisions.

To date, most economic evaluations on TYRX have focused on patients at increased risk of infection.^1,13,14^ Previous cost-effectiveness analyses conducted across different healthcare systems, including those of the United States, United Kingdom, Germany, Italy, and Denmark, have consistently shown that TYRX is cost-effective in patients at elevated risk for device-related infections, with ICERs below commonly accepted thresholds in all settings examined.^1,13,14,33^ Additionally, Kay et al included an analysis in an unselected population and found that TYRX remained cost-effective in this broader group at a £30 000/QALY-gained threshold.^13^ Although the populations and healthcare settings differ, the findings of the present study are consistent with this body of evidence.

While these studies have consistently demonstrated favorable cost-effectiveness, their applicability to broader clinical practice has been limited. The present analysis, by accounting for the full spectrum of patients receiving CIEDs in routine care, provides valuable insights for hospitals that may be considering broader implementation of infection prevention strategies, and challenges the notion that antibacterial envelopes should be reserved exclusively for high-risk cases.^34^

To our knowledge, this study is the first economic evaluation of TYRX in Spain and provides novel evidence in a broad, unselected population. The results show that, by reducing infection rates and avoiding their downstream consequences, TYRX is highly likely to be cost-effective when used in this wider population.

An alternative scenario, which evaluates the results in a high-risk population, showed even more favorable outcomes. This is consistent with expectations, as higher-risk patients derive greater benefit from infection prevention strategies. Nonetheless, these findings continue to reinforce the broader application of antibacterial envelopes, with the understanding that their value increases proportionally with infection risk.

When evaluating by device type, TYRX demonstrated favorable results across the spectrum of CIEDs, with particularly strong outcomes in recipients of high-power devices (CRT-D and ICD). This was primarily driven by higher infection-related expenses associated with the greater device cost and subsequent replacements. In patients receiving low-power devices (PPM and CRT-P), TYRX was also found to be cost-effective, providing clear clinical benefit at a reasonable incremental cost.

Therefore, the evidence shows that TYRX is likely to be a cost-effective strategy for infection prevention across diverse populations and device categories, emphasizing its value in routine clinical practice. Moreover, the results demonstrate that even considering a shorter time horizon of 3 years, TYRX remains cost-effective across all devices, which highlights its potential to deliver economic and clinical benefits not only in the long-term, but also in the short-term.

There are limitations in the present model that should be considered when interpreting the results. Many clinical inputs were sourced from studies conducted outside Spain due to limited availability of local data, which may affect generalizability. Specifically, infection rates were obtained from the REINFORCE study, a real-world Italian cohort considered broadly comparable to the Spanish setting.^12^ Although not a randomized trial, REINFORCE provided data from a propensity-matched analysis and was preferred due to the potential underestimation of infection rates in clinical trials caused by the Hawthorne effect, where awareness of observation may lead to improved care and lower event rates.^12,35^ In the absence of country-specific data for Spain, European and international consensus documents, including the EHRA International Consensus on CIED Infection Prevention and Management,^9^ provide widely accepted standards for prophylaxis and perioperative infection control practices that are generally implemented across hospitals in Italy and Spain. These shared practices support the applicability of REINFORCE infection rates to the Spanish context, while acknowledging that subtle differences in hospital organization or patient characteristics may exist. Nevertheless, the model would benefit from future updates incorporating Spanish-specific data. Limitations also stem from the theoretical nature of cost-effectiveness modeling, which may not fully reflect clinical practice. For instance, regarding CIED-related infections, variability in hospital protocols and resource use can influence both infection rates and costs. Additionally, the lack of device-specific infection and mortality rates required applying uniform estimates across all CIED types. Lastly, in the high-risk scenario, several assumptions were made due to data gaps: the distribution and timing of infections, the treatment effect of TYRX, and mortality rates were all assumed to be the same as in the broad, unselected population, which may have led to overestimated survival in this subgroup. In addition, baseline infection risk was estimated using data from a separate study. Despite these limitations, all model inputs and assumptions were validated by a panel of clinical experts, and sensitivity analysis confirmed the robustness of the results.

## CONCLUSIONS

The results of the present model suggest that the use of TRYX for preventing CIED infections could be considered a dominant strategy for CRT-D and ICD devices, and a cost-effective intervention for CRT-P and PPM when assessed against the commonly accepted willingness-to-pay threshold in Spain. Its widespread implementation could contribute to significant clinical benefits and more efficient resource use within the Spanish National Health System.

### Disclosures

T.D. and A.d.A. received consultancy fees from Medtronic for their participation in the analysis. A.d.A. has participated in scientific symposiums sponsored by Medtronic and has also received funding for research studies on infections related to cardiac electrostimulation devices. He is also a proctor for Cook Medical for percutaneous device removal and has received funds for investigational purposes. D.A., E.G., and S.J.M. are employees of York Health Economics Consortium, who were commissioned by Medtronic to provide consultancy and write the manuscript. M.A., J.M., S.E., and C.W. are full-time employees of Medtronic. The authors hereby declare that this economic support has not interfered with the development of this project and that the sponsor did not participate in or influence the analysis of the present study or interpretations of its results.
